# Differential modulation of lung aquaporins among other pathophysiological markers in acute (Cl_2_ gas) and chronic (carbon nanoparticles, cigarette smoke) respiratory toxicity mouse models

**DOI:** 10.3389/fphys.2022.880815

**Published:** 2022-09-28

**Authors:** Sukanta S. Bhattacharya, Brijesh Yadav, Ekta Yadav, Ariel Hus, Niket Yadav, Perminder Kaur, Lauren Rosen, Roman Jandarov, Jagjit S. Yadav

**Affiliations:** ^1^ Pulmonary Pathogenesis and Immunotoxicology Laboratory, Department of Environmental and Public Health Sciences, University of Cincinnati College of Medicine, Cincinnati, OH, United States; ^2^ Department of Physiology and Biophysics, Case Western Reserve University School of Medicine, Cleveland, OH, United States; ^3^ Department of Biology, University of Miami, Coral Gables, FL, United States; ^4^ Medical Scientist Training Program, University of Virginia School of Medicine, Charlottesville, VA, United States; ^5^ Department of Pathology and Laboratory Medicine, University of Cincinnati, UC Health University Hospital Laboratory Medicine Building, Cincinnati, OH, United States; ^6^ Division of Biostatistics and Bioinformatics, Department of Environmental and Public Health Sciences, University of Cincinnati College of Medicine, Cincinnati, OH, United States

**Keywords:** aquaporin, chlorine gas, carbon nanotubes, cigarette smoke extract, gene expression, lung toxicity

## Abstract

Inhaled toxic chemicals and particulates are known to disrupt lung homeostasis causing pulmonary toxicity and tissue injury. However, biomarkers of such exposures and their underlying mechanisms are poorly understood, especially for emerging toxicants such as engineered nanoparticles and chemical threat agents such as chlorine gas (Cl_2_). Aquaporins (AQPs), commonly referred to as water channels, are known to play roles in lung homeostasis and pathophysiology. However, little is known on their regulation in toxicant-induced lung injuries. Here, we compared four lung toxicity models namely, acute chemical exposure (Cl_2_)-, chronic particulate exposure (carbon nanotubes/CNT)-, chronic chemical exposure (cigarette smoke extract/CSE)-, and a chronic co-exposure (CNT + CSE)- model, for modulation of lung aquaporins (AQPs 1, 3, 4, and 5) in relation to other pathophysiological endpoints. These included markers of compromised state of lung mucosal lining [mucin 5b (MUC5B) and surfactant protein A (SP-A)] and lung-blood barrier [protein content in bronchoalveolar lavage (BAL) fluid and, cell tight junction proteins occludin and zona-occludens]. The results showed toxicity model-specific regulation of AQPs measured in terms of mRNA abundance. A differential upregulation was observed for AQP1 in acute Cl_2_ exposure model (14.71-fold; *p* = 0.002) and AQP3 in chronic CNT exposure model (3.83-fold; *p* = 0.044). In contrast, AQP4 was downregulated in chronic CSE model whereas AQP5 showed no significant change in any of the models. SP-A and MUC5B expression showed a decreasing pattern across all toxicity models except the acute Cl_2_ toxicity model, which showed a highly significant upregulation of MUC5B (25.95-fold; *p* = 0.003). This was consistent with other significant pathophysiological changes observed in this acute model, particularly a compromised lung epithelial-endothelial barrier indicated by significantly increased protein infiltration and expression of tight junction proteins, and more severe histopathological (structural and immunological) changes. To our knowledge, this is the first report on lung AQPs as molecular targets of the study toxicants. The differentially regulated AQPs, AQP1 in acute Cl_2_ exposure versus AQP3 in chronic CNT nanoparticle exposure, in conjunction with the corresponding differentially impacted pathophysiological endpoints (particularly MUC5B) could potentially serve as predictive markers of toxicant type-specific pulmonary injury and as candidates for future investigation for clinical intervention.

## Introduction

The rising trend in industrialization and technological advancements in the modern world has led to improved human life but not without its challenges to environmental and human health. The human respiratory system is routinely exposed to toxic chemicals and particulates, often as chronic exposures (such as to ambient or indoor air pollution, occupational environment, among others) (Walker et al., 2022; Wang et al., 2022) but occasionally as acute exposures (such as *via* industrial accidents, spills, and chemical threats or warfare) (Mishra et al., 2009; Razavi et al., 2013). Particularly, the emerging high-volume nanomaterials such as carbon nanotubes pose respiratory risks *via* chronic occupational or environmental exposures during the product life cycle (Beard et al., 2018; Guseva Canu et al., 2020). Such environmental exposures may be compounded by chronic coexposure from unhealthy lifestyle practices, particularly smoking (Morissette et al., 2014; Dai et al., 2021; Yang et al., 2021). On the other hand, highly toxic gases such as chlorine gas (Cl_2_) (Addis et al., 2020; Lazrak et al., 2020) have been misused as chemical threat agents and in chemical warfare because of their acute toxicity to respiratory system (Govier and Coulson, 2018; Jacobs and Kovac, 2020).

Inhaled toxicants may variably induce lung pathophysiology causing toxicity/injury-associated acute conditions (such as pulmonary edema, acute respiratory distress syndrome) and/or chronic lung diseases such as asthma, chronic obstructive pulmonary disease (COPD), pulmonary fibrosis, among others (Frank et al., 2016; Addis et al., 2020; Lazrak et al., 2020). Pathophysiological changes from acute and chronic toxic exposures are often characterized by inflammation, edema, fibrosis, and/or hyperplasia/dysplasia. However, the knowledge on the underlying cellular and molecular targets governing the pathophysiological phenotypes of pulmonary toxicity is still evolving, particularly for the emerging toxicants and chemical threat agents. While majority of the toxicological studies have targeted innate protective components governing lung plasticity or defense (such as surfactant proteins, mucins) as well as breach of airway-blood barrier (cell junction proteins), role of water channels (aquaporins) in toxicity mechanisms is poorly understood. The critical roles aquaporins are known to play in homeostasis and pulmonary pathophysiology make them interesting target candidates for understanding their role in lung toxicity mechanisms (Song et al., 2000; Yadav et al., 2020).

Aquaporins (AQPs) are a family of hydrophobic, integral membrane proteins expressed on plasma membranes in several cell types. These small protein channels are known to play a major role in osmotically-driven transcellular and transepithelial water movement (Kreda et al., 2001; Woo et al., 2008) across cell membranes. As of today, mammals have been shown to possess 13 types of related AQPs, of which at least four (AQPs 1, 3, 4, and 5) are expressed in lung. Of these lung aquaporins, AQP1 and AQP5 are primarily expressed in vasculature endothelia and alveolar epithelia respectively to allow for movement of the fluid in the alveoli between the air space and the associated vasculature. AQP3 is expressed in the basal epithelial cells of the nasopharynx and large airways, whereas AQP4 is expressed in the ciliated columnar epithelial cells of nasopharynx, trachea, and bronchus (Yadav et al., 2020). The lung AQPs have been shown to be responsive to acute lung injury (ALI), particularly that associated with oxygen levels (ventilator use, high altitude) (Hales et al., 2001; Fabregat et al., 2016) and infections (Vassiliou et al., 2013; Zhang et al., 2018; Creed et al., 2020; Allnoch et al., 2021). Aquaporins have also been reported to modulate lung injury or related pathophysiology in animal models. For instance, AQP3 was reported to potentiate ovalbumin-induced asthma (Ikezoe et al., 2016). Hypoxia-induced pulmonary hypertension which upregulated AQP1 was reversed by targeting this AQP. The idea of therapeutic targeting of aquaporins is receiving increasing focus for attenuation of various forms of pathologies including induced lung injuries (Guo et al., 2020; Li et al., 2020; Moosavi and Elham, 2020). However, little is known on regulation of lung aquaporins in toxicant-induced lung injuries and their relevance in acute versus chronic toxic exposures.

In this investigation using inbred mice, we focused on three toxicants (carbon nanotubes, cigarette smoke chemicals mix, and Cl_2_ gas). Based on their common mode of exposure (acute versus chronic) in the real world, the mice were subjected to either acute or chronic exposure regime to design four distinct lung toxicity models. These included an acute chemical exposure model (mice exposed to Cl_2_ gas), a chronic particle exposure model [mice exposed to multiwalled carbon nanotubes (CNT)], a chronic chemical exposure model [mice exposed to cigarette smoke extract (CSE) mimicking smoking in humans], and a chronic hybrid/coexposure model (mice exposed to CNT nanoparticles and CSE chemicals). The models were evaluated for the relevance of lung aquaporins (AQPs 1, 3, 4, and 5) and other homeostasis-associated constituents/markers namely, mucin 5B (MUC5B), surfactant protein A (SP-A), and cell tight junction proteins occludin (OCLN) and zona-occludens 1 (ZO-1) in lung injury and the ensuing pathogenesis in the toxicant-exposed lungs. Given that the three toxicant types selected in this study are known to induce distinct pathophysiological phenotypes in the exposed lungs, molecular targets differentially modulated by these toxicant types may potentially reveal biomarkers of exposure; these may be useful in diagnosis and/or prognosis as well as timely triaging for clinical intervention, particularly in idiopathic or acute toxicity situations including chemical threat or warfare scenarios. For instance, mass casualty and disaster victims acutely exposed to chemical threat and warfare agents such as Cl_2_ may require biomarkers to predict the nature of exposure. Likewise, the survivors may be triaged to receive immediate clinical intervention based on such biomarkers of exposure. On the other hand, specific biomarkers of chronic toxicant exposure may help identify those at long-term risk of developing lung conditions/diseases and may enable timely intervention.

In the current study, we exposed mice to the individual study toxicants and evaluated their impact on lung aquaporins and other protein targets, particularly those with a role in lung innate protection/function and integrity as well as lung immuno-pathology.

## Materials and methods

### Ethics statement

The mice exposure experiments were conducted at the Laboratory Animal Medical Services facility of the University of Cincinnati. All animal protocols were approved by the University’s Institutional Animal Care and Use Committee (IACUC).

### Animals and toxicant exposures

C57BL/6J mice (6–8 weeks-old), generated by in-house breeding, were used in the entire study. The mice were divided into six cohorts (*n* = 4 in each cohort) and subjected to the following four toxicant treatments: inhalation exposure to Cl_2_ gas at 200 ppm for 5 min; one-time dosing (2.8 mg/kg body weight) with a suspension of multiwall carbon nanotubes (abbreviated as CNT throughout this document) *via* oropharyngeal instillation on day 1 for a 4-week exposure; daily dosing (30 µl) with cigarette smoke extract (CSE) administered *via* oropharyngeal instillation for 4 weeks; dosing with a combination of the above CNT and CSE regimens. Control mice groups were instilled with the vehicle or filtered air (in case of Cl_2_ gas-treated mice) using the same exposure period for comparisons.

### Carbon nanotubes preparation and characterization

High-purity multi-wall carbon nanotubes (Baytubes®), were obtained from Bayer MaterialScience company (Leverkusen, Germany) in a dry bulk powder form. These were processed to prepare a uniform suspension suitable for oropharyngeal instillation in mice as per the protocol described in our previous reports (Frank et al., 2015; Frank et al., 2016).

### Cigarette smoke extract preparation

Reference cigarettes (Code 3R4F) were procured from the Center for Tobacco Reference Products, Kentucky Tobacco Research and Development Center, Lexington, Kentucky, United States. For preparation of cigarette smoke extract (CSE), we used a modified in-house method based on a previous report for the preparation of such extract (Higashi et al., 2014). Briefly, the smoke emitting from 10 ignited cigarettes was vacuum-drawn through a customized Cigarette Smoke Extractor set-up Supplementary Figure S1 and passed through sterile PBS (10 ml) for 10 min. The extract was aseptically collected and stored in airtight vials at 4°C for further use.

### Tissue and fluid harvesting

The mice were sacrificed for various analyses by intraperitoneal injection with Euthasol^®^ (Butler-Schein, Dublin, OH). Bronchoalveolar Lavage (BAL) fluid was collected after perfusion with Phosphate Buffered Saline (PBS) solution. For gene expression analysis, intact lungs were harvested, snap frozen in liquid N_2_, and stored in −80°C for total RNA isolation. For histopathological analysis, the lungs were fixed in 10% paraformaldehyde solution overnight at 4°C.

### RNA isolation

Total RNA was isolated from the mouse lungs using a combination of commercial kits. First, TRI reagent kit (Molecular Research Center, Cincinnati, OH) was used to extract total RNA from the lung homogenate as per the manufacturer’s protocol. The crude RNA extract (recovered from the aqueous phase) was then purified in a column-based protocol using RNeasy® mini kit (Qiagen). The RNA preparation was checked for quality and concentration using Nanodrop ND-2000c (Nunc Nanodrop, Thermofisher, United States).

### Quantitative reverse transcription-polymerase chain reaction for gene expression analysis

Gene-specific quantitative reverse transcription-polymerase chain reaction (RT-PCR) was performed using Brilliant III SYBER Green Master Mix one-step kit (Agilent Technologies, Santa Clara, CA) and real-time PCR platform ABI 7500 (Applied Biosystems). Gene-specific primers used in this study were custom synthesized by Integrated DNA Technologies Inc., Coralville, IA. Table 1 gives the primer sequences and their PCR efficiency values. All primer pairs were tested for amplification efficiency based on the formula published elsewhere (Svec et al., 2015) and using the online efficiency calculator tool of Thermofisher Scientific, Waltham, MA (Thermofisher Scientific, 2022). The following gene targets were tested using glyceraldehyde-3-phosphate dehydrogenase (GAPDH) as a house-keeping control gene: aquaporin 1 (AQP1), aquaporin 3 (AQP3), aquaporin 4 (AQP4), aquaporin 5 (AQP5), surfactant protein A (SP-A), mucin 5b (MUC5B), occludin (OCLN), and zona-occludens 1 (ZO-1). Since AQP4 gene is known to have variants, we used global BLAST search criteria to ensure that the designed primer pair shows 100% match with all mouse gene variants available in the database. One-step qRT-PCR reaction for each gene target was performed in triplicate using RNA template (10 ng) in a defined reaction volume (10 µl). The amplification conditions were as follows: reverse transcription step at 50°C for 10 min followed by denaturation at 95°C for 10 min, and 40 cycles of amplification (each cycle involving denaturation at 95°C for 15 s and amplification at the respective optimized target-specific annealing temperature for 60 s). Raw cycle threshold (CT) values of the target gene (TG) were normalized against the housekeeping gene (HKG) GAPDH. Fold change (FC) in target gene expression in the sample relative to the control was calculated (Schmittgen and Livak, 2008). The calculation was based on the formula: Fold Change = 2^−[Ct (TG)-Ct (HKG)] treated sample—mean of [Ct (TG)-Ct (HKG) control group] where TG is the target gene and HKG is the housekeeping gene. For the downregulated genes, where the calculated value based on the above formula is <1, fold change was expressed as the negative reciprocal of the value (Schmittgen and Livak, 2008).

**TABLE 1:** Primers used in the present study. All the primers were ordered from Integrated DNA Technologies Inc., Coralville, IA, United States and tested in-house for primer efficiency (represented as % Mean ± SD) which ranged between the acceptable limits (90%–110%).

Target gene	Forward primer (5′-3′)	Tm (°C)	Reverse primer (5′-3′)	Tm (°C)	Primer efficiency (%)
GAPDH	CAC CCT TCA AGT GGG CCC CG	63.5	TTG GCC GTA TTG GGC GCC TG	63.4	104.7 ± 1.2
AQP1	CTT TGG TCT GAC TTA CCT CCA G	54.8	TCC CTT CCT TGC CAC TTT AC	54.8	98.2 ± 0.5
AQP3	TGG AAT CTT TGC CAC CTA TCC	54.7	TGG CCA GTA CAC ACA CAA TAA	54.2	103.1 ± 2.7
AQP4	GGT AAT GGA GGG CTT TCT TCT C	55.2	GCA CCG AAG AGA ATC AGG TTT A	54.8	107.9 ± 1.8
AQP5	GAC AGG GCC TCT TTG GAA TTA	54.7	CTG GGT TGG AGA GAT AAA CAG G	54.9	103.2 ± 4.5
SP-A	GTA TTC TCG GCT GTA CCT GCC	57.5	GAG GTC CAG GGT CTC CTT TGA	58.3	108.9 ± 1.1
MUC5B	GAG GTC AAC ATC ACC TTC TGC	55.8	TCT CAT GGT CAG TTG TGC AGG	57.2	95.9 ± 0.1
ZO-1	AGC CTG CAA AGC CAG CTC A	60.2	AGT GGC CTG GAT GGG TTC ATA G	58.9	96.6 ± 4.1
OCLN	CTT TGG CTA CGG AGG TGG CTA T	59	CTT TGG CTG CTC TTG GGT CTG	58.5	94.7 ± 4.2

### Total protein in bronchoalveolar lavage fluid

BAL protein content was determined using DC protein estimation kit (BioRad, Hercules, CA) following the manufacturer’s instructions.

### Histopathology of lung tissue

The formalin-fixed whole lungs were processed for histopathological analysis at the University’s Children’s Hospital and Medical Center (CCHMC) core facility, with histopathologic image analysis performed at the University of Cincinnati Medical Center. Hematoxylin and Eosin (H&E) staining was performed using standard approach. A blinded histopathological analysis was performed using Olympus BX53 microscope equipped with Olympus DP22 and the software system cellSens (Olympus). The following architectural and immunologic pathology parameters were examined: occurrence of interstitial widening, interstitial inflammation (monocytic or neutrophilic), intra-alveolar immune infiltration (alveolar macrophages, neutrophils, and other immune cell forms), granulomas, vascular congestion, edema (intra-alveolar or perivascular). Treated lung sections were scored for these features with respect to the control lungs on a 0 to 4 scale, with 0 = no change, 1 = minimal change, 2 = mild change, 3 = moderate change, and 4 = severe change. When compared to the control lungs, “no change” implied normal or unchanged lung in terms of a given histological feature, whereas the minimal, mild, moderate, and severe changes implied increasing gradation of change in terms of a given histological feature.

### Statistical analyses

All statistical analyses were performed using GraphPad Prism 9.3.1 (La Jolla, CA, United States). We carried out unpaired *t*-tests with Welch’s correction to investigate the average difference in each outcome for only the pairs of groups targeted for this study. For each outcome, therefore, we performed four *t*-tests (CNT vs. vehicle control, CSE vs. vehicle control, CNT + CSE vs. vehicle control, and Cl_2_ gas vs. air control). To account for multiple comparisons, we applied the conservative Bonferroni correction, and all raw *p*-values were increased by a factor of 4. Our null hypotheses for *t*-tests were that there is no difference in outcome between the given two groups. Alternative hypotheses for the *t*-test were that the outcome, on average, is different between the given two groups. Power calculations were performed for all the t-tests and our results confirmed that we had statistical power close to or over 80% (75%–100%) for all the key significantly impacted targets. Adjusted *p*-values ≤ 0.05 were considered statistically significant. All *p*-values representing statistical significance have been displayed on the corresponding plots.

## Results

Lung exposure to the individual study toxicants caused altered transcriptional expression of lung aquaporins as well as other proteins responsible for lung homeostasis (MUC5B and SP-A) and tissue integrity (OCLN and ZO-1) in the respiratory system.

### Expression levels of lung aquaporins were differentially altered in different toxicity models

**FIGURE 1:**
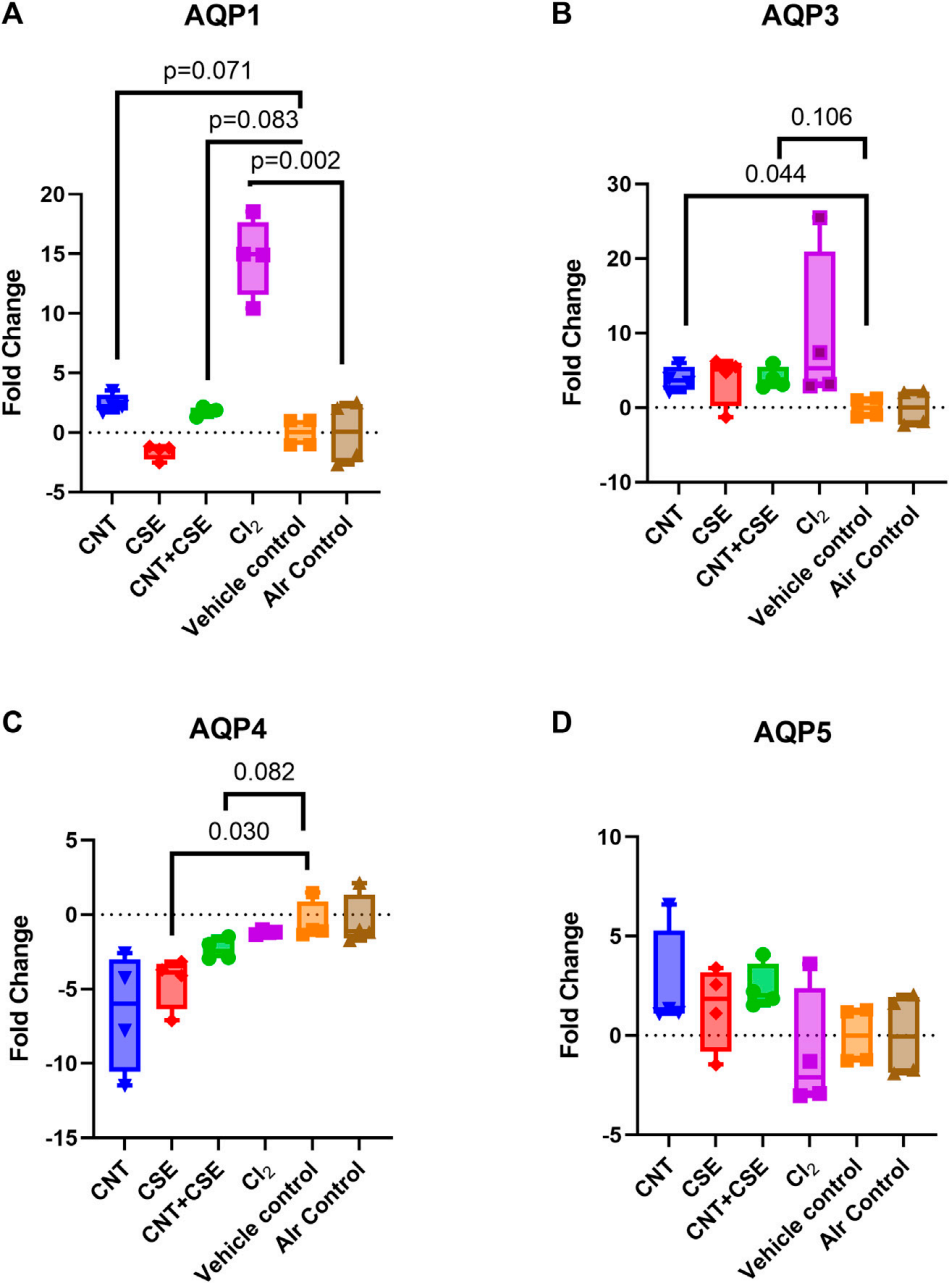
Expression of lung aquaporins [AQP1 **(A)**, AQP3 **(B)**, AQP4 **(C)**, and AQP5 **(D)**] in mice subjected to different respiratory exposure regimens namely, acute chemical exposure to Cl_2_ gas, chronic particulate exposure to carbon nanotubes (CNT), chronic chemical exposure to cigarette smoke extract (CSE), and a chronic co-exposure to CNT + CSE. Mice (6–8 weeks-old) were exposed *via* inhalation to Cl_2_ gas (@200 ppm) for 5 min and *via* oropharyngeal administration to CNT suspension (2.8 mg/kg body weight single dose on day 0), CSE suspension (30 µl/mouse daily), or the combination regimen (CNT + CSE) for a duration of 28 days. Total RNA of the lung tissue from each mouse was subjected to qRT-PCR based gene expression analysis for individual aquaporins (AQPs 1, 3, 4, and 5) represented as fold change, as described under Materials and Methods section. Statistical differences are represented by *p*-values.

Acute exposure to Cl_2_ gas and chronic exposure to CNT, CSE, or CNT + CSE were compared for transcriptional expression of all four lung aquaporins (AQPs 1, 3, 4, and 5), relative to the controls, expressed as fold changes in mRNA abundance (Figure 1). Differential changes were observed across the treatment groups in the expression of AQP1 (Figure 1A), AQP3 (Figure 1B), and AQP4 (Figure 1C), with no appreciable change in AQP5 (Figure 1D). AQP1 showed significant upregulation (14.71-fold, *p* = 0.002) in the acute Cl_2_ toxicity model. In contrast, chronic toxicity models showed non-significant increase [CNT (2.411-fold, *p* = 0.071), and CNT + CSE (1.778-fold, *p* = 0.083)] or decrease [CSE (−1.594-fold, *p* = 0.269)]. AQP3 showed significant upregulation in CNT toxicity model (3.83-fold, *p* = 0.044), unlike the other models which did not reach statistical significance (Cl_2_ 9.73-fold, *p* > 0.2, CNT + CSE = 3.93 fold, *p* = 0.106 and CSE 3.77-fold, *p* > 0.2), implying its differential responsiveness to CNT exposure. AQP4 was significantly downregulated in the CSE toxicity model (−4.515-fold, *p* = 0.030) unlike Cl_2_, CNT, and CNT + CSE models which showed a non-significant decrease in expression. AQP5 did not show significant change in any of the toxicity models.

### Mucosal homeostasis and defense proteins (mucin 5B and surfactant protein A) showed variable transcriptional expression in different toxicity models

**FIGURE 2:**
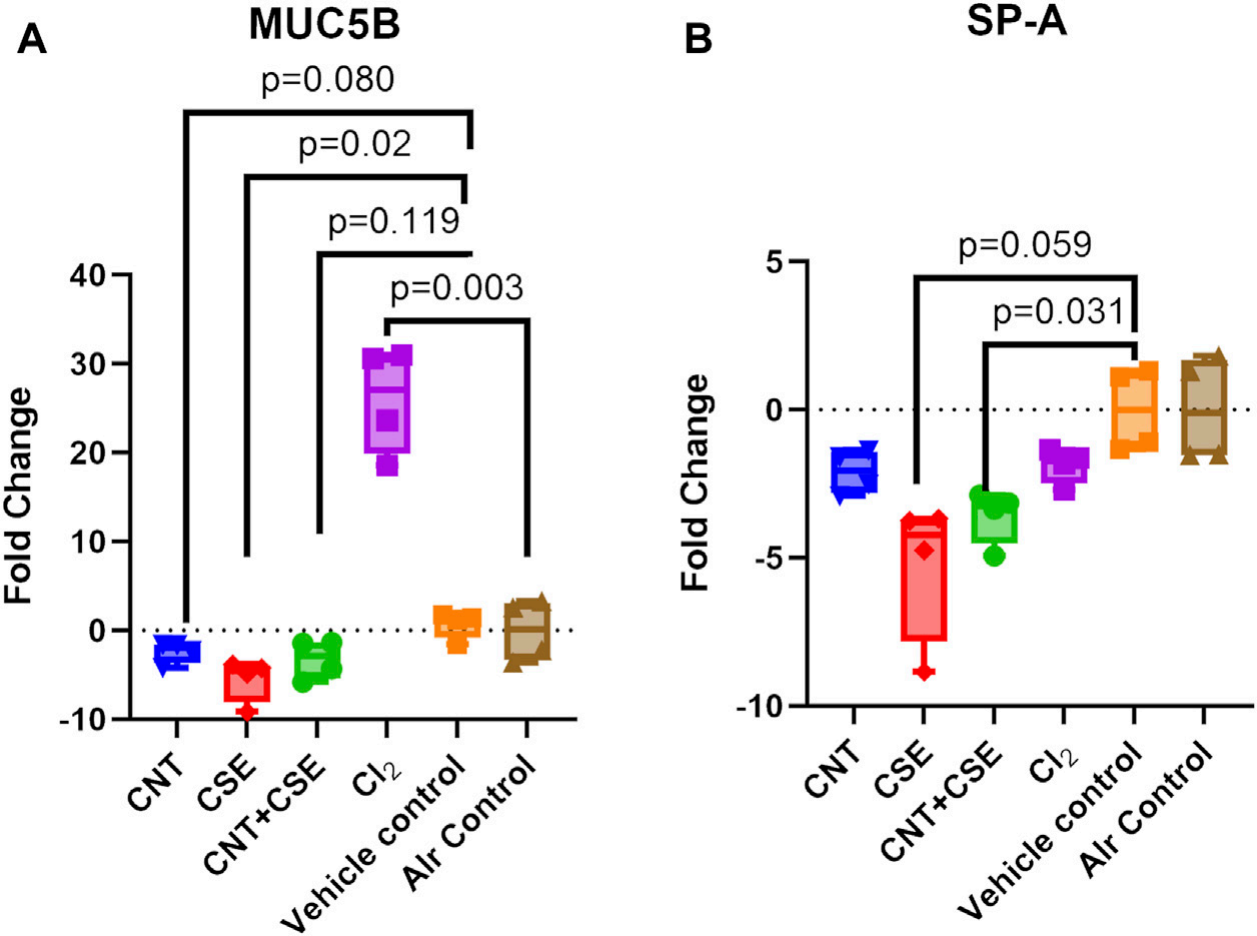
Expression of lung mucosal defense proteins [MUC5B **(A)** and SP-A **(B)**] in mice subjected to different respiratory exposure regimens for CNT, CSE, CNT + CSE, and Cl_2_, as described in the Figure 1 legend. Transcriptional expression of the target proteins was measured using gene-specific qRT-PCR. Statistical differences are represented by *p*-values.

Lung MUC5B (Figure 2A) and SP-A (Figure 2B) showed variable transcriptional response to the different toxicity models investigated in this study. While acute Cl_2_ toxicity model showed high significant upregulation of MUC5B expression (25.95-fold, *p* = 0.003), chronic CSE toxicity model (−5.506-fold, *p* = 0.028) showed a significant downregulation. A decrease, though statistically non-significant, was also observed in CNT (−2.409-fold, *p* = 0.080) and CNT + CSE (−3.232-fold, *p* = 0.119) models. On the other hand, SP-A expression showed downregulation in CNT + CSE (−3.573-fold, *p* = 0.031) and CSE (−5.250 fold, *p* = 0.059) toxicity models. CNT and Cl_2_ models, however, did not show significant impact on the SP-A expression. These findings collectively imply a significant dominant effect of cigarette smoke on SP-A expression.

### Airway-blood barrier integrity markers were differentially expressed in different toxicity models

**FIGURE 3:**
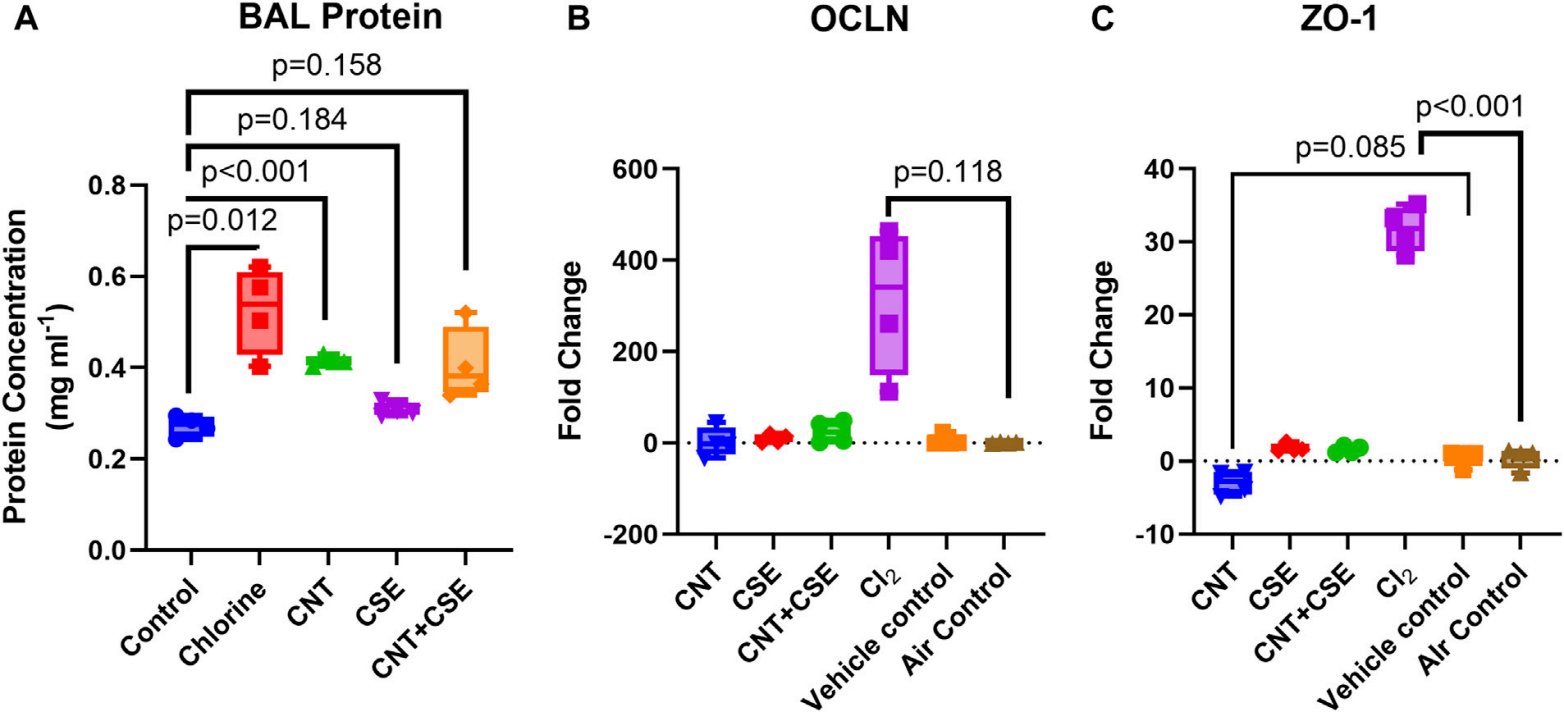
Quantitative assessment of compromised lung integrity based on protein filtration [BAL protein content **(A)**] and expression of cell tight junction proteins [OCLN **(B)** and ZO-1 **(C)**] in mice subjected to different respiratory exposure regimens for CNT, CSE, CNT + CSE, and Cl_2_, as described in the Figure 1 legend. Transcriptional expression of the cell tight junction proteins occludin (OCLN) and zona-occludens-1 (ZO-1) was measured using gene-specific qRT-PCR. Statistical differences are represented by *p*-values.

Effect on airway epithelial-endothelial barrier integrity was evaluated based on the total protein content in the BAL fluid (Figure 3A) and the mRNA expression levels of cell tight junction proteins OCLN (Figure 3B) and ZO-1 (Figure 3C).

A significant increase in the BAL fluid protein content was observed in the Cl_2_ toxicity model when compared to the control (0.525 mg·ml^−1^ in exposed versus 0.272 mg·ml^−1^ in control lungs, *p* = 0.012). The chronic toxicity models also showed an increased BAL protein content. While CNT model showed a highly significant increase (0.414 mg·ml^−1^, *p* < 0.001), the increase of BAL protein content in CSE (0.308 mg·ml^−1^, *p* = 0.184) and CNT + CSE (0.406 mg·ml^−1^, *p* = 0.159, Welch’s *t*-test) models was not statistically significant.

The different toxicity models showed variable trends in transcriptional expression of the tight junction proteins ZO-1and OCLN. Notably, ZO-1 was significantly overexpressed in the Cl_2_ exposed mice (31.73-fold, *p* < 0.001) but showed no change in chronic toxicity models with the exception of CNT model which showed a decreased expression (−2.952-fold, *p* = 0.085). For OCLN, the acute Cl_2_ toxicity model showed a very high fold-increase in the mean expression (314.4-fold), though without statistical significance (*p* = 0.118). The chronic toxicity models showed no significant impact on OCLN expression.

### Histopathological changes in toxicant-exposed lungs

**FIGURE 4:**
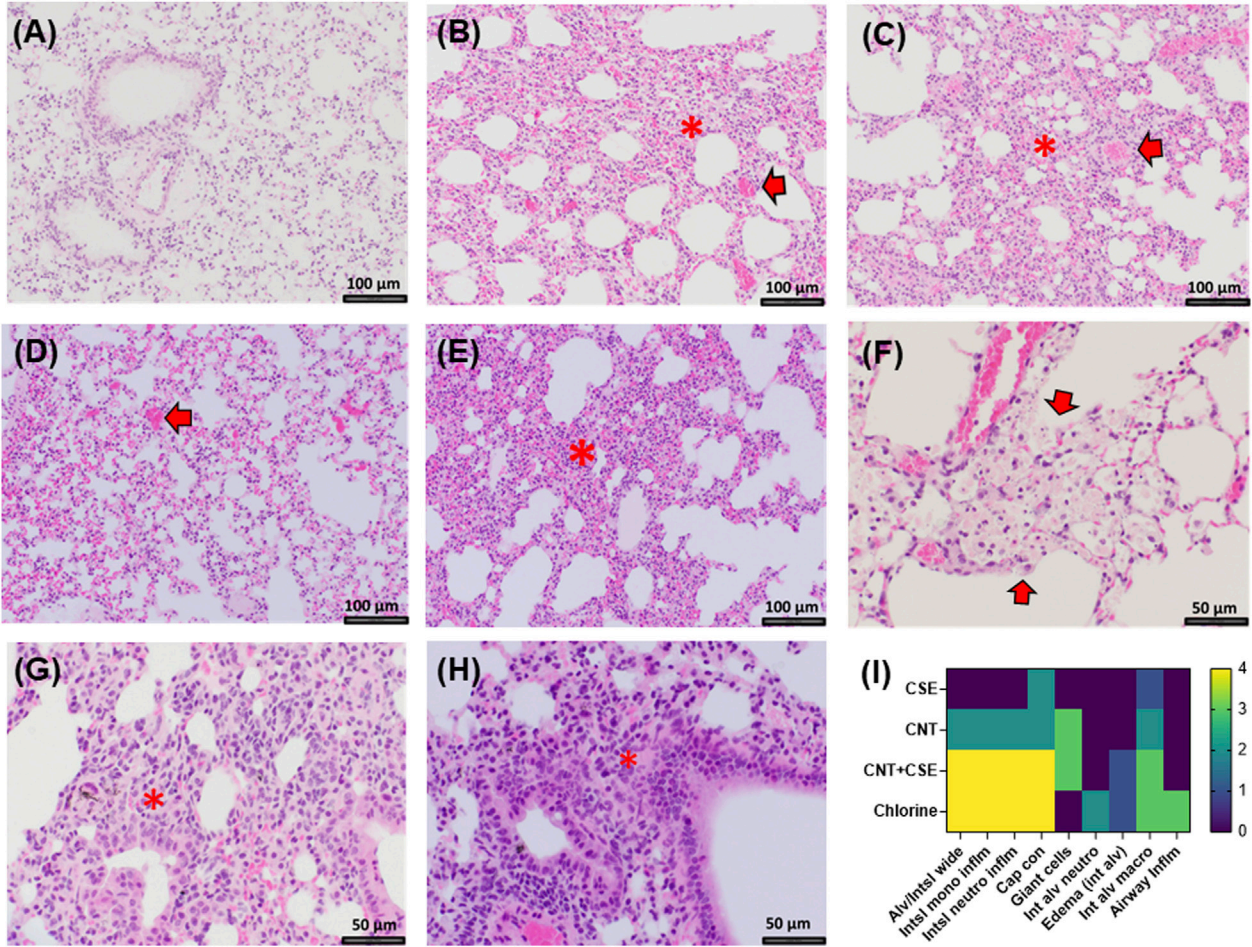
Histopathological changes in the lungs of mice exposed to different toxicants: **(A).** Control mice (×200 magnification); **(B).** Mice exposed to Cl_2_ showing interstitial widening (*) and capillary congestion (arrow, ×200 magnification); **(C).** Mice exposed to CNT showing patchy vascular congestion (arrow) and interstitial widening (*, ×200 magnification); **(D).** Mice exposed to CSE showing capillary congestion (arrow, ×200 magnification); **(E).** CNT + CSE coexposed mice showing interstitial widening (*, ×200 magnification); **(F).** Cl_2_ exposed mice showing intra-alveolar foamy macrophages and debris material (arrows, ×400 magnification); **(G).** Granulomatous reaction in CNT exposed lungs (*, ×400 magnification); **(H).** Peribronchiolar granulomatous reaction in lungs coexposed to CNT and CSE (*, ×400 magnification) **(I).** Heat map (scored as 0 to 4 on scale, with 0 being normal and 4 being severe) showing the effect of different exposures on different immuno-histological parameters. Abbreviations: Alveolar/Interstitial widening (Alv/Intsl wide); Interstitial mononuclear inflammation (Int/mononu Inflm); Interstitial neutrophil inflammation (Int neut inflm); Capillary congestion (Cap con); Giant cells, Intra-alveolar neutrophils (Int alv neutro); Intra-alveolar edema (edema int alv); Intra-alveolar Macrophages (Int alv macro); Airway inflammation (Airway inflm). Magnification scale is depicted with a size-labelled bar on individual images **(A–H)**.

Cl_2_ exposed lungs showed diffuse interstitial widening due to capillary congestion and interstitial cellular infiltrate (monocytes and neutrophils) and accumulation of foamy macrophages and debris material within peribronchiolar air spaces (Figure 4). A similar pattern of diffuse interstitial widening due to capillary congestion and increased interstitial inflammation was seen in the CNT + CSE group. In comparison, CNT group showed milder patchy interstitial widening. Both CNT and CNT + CSE exposed lungs showed granulomatous reaction to CNTs within a peribronchiolar distribution. CSE exposed lung showed patchy mild capillary congestion and diffuse perivascular edema. Rare foci of intra-alveolar edema were present in both Cl_2_ and CNT + CSE exposed lungs.

## Discussion

**FIGURE 5:**
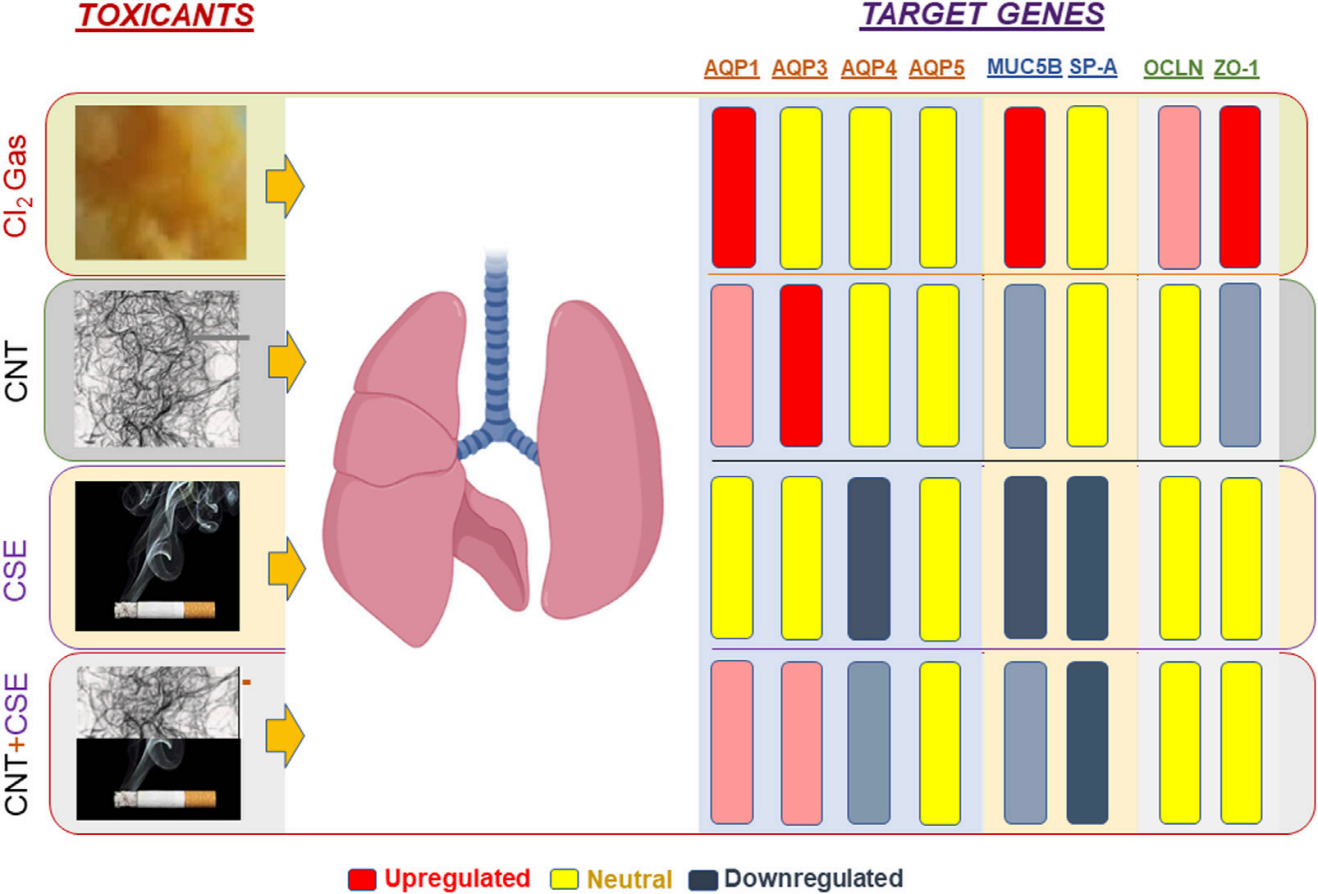
Schematic representation of toxicant-specific target gene expression patterns in exposed mouse lungs. The toxicants tested are shown on the left-hand side of the lungs whereas the target genes quantified are shown on the right-hand side. Gene regulation pattern is color coded as follows: Upregulation (red), Downregulation (blue-gray), Neutral or Unchanged/Statistically non-significant (yellow). The extent of regulation (fold change) is reflected by the color intensity.

Using four distinct models of lung toxicity/injury induced by three toxicants (Cl_2_, CNT, CSE), this study compared modulation of lung aquaporins (namely AQPs 1, 3, 4, and 5) and other lung structural proteins with known role in innate protective and homeostatic function (MUC5B and SP-A protein) as well as the markers indicative of a compromised lung epithelial-endothelial cell junction integrity (BAL protein content and cell junction proteins OCLN and ZO-1). The results revealed three major differential outcomes. First, the data supported our hypothesis that lung aquaporins are differentially modulated in a toxicant-specific manner. Second, the major gel-forming mucin of lung mucosa MUC5B is significantly upregulated in the acute chemical toxicity model (Cl_2_), while showing a decreasing pattern in the chronic chemical/particulate toxicity models. Third, the acute Cl_2_ toxicity model triggered highly elevated expression of the cell tight junction proteins particularly ZO-1, unlike the chronic toxicity models (Figure 5). Likewise, the Cl_2_ toxicity model showed significant infiltration of proteins in the lung lumen unlike the chronic models except the CNT model. Measurement of protein abundance will provide further validation for these mRNA abundance-based patterns for the tight junction proteins.

Among the aquaporins, AQP1 was significantly upregulated (in terms of mRNA abundance) in the acute toxicity model (Cl_2_). Such significant overexpression was not observed in any of the chronic toxicity models. Lung AQP1 is known to be expressed in the endothelial cells, fibroblasts, and mesothelial cells and has been associated with rapid water movement by transmembrane gradient (Tsunoda et al., 2004), as has been shown in hydrostatically-induced lung interstitial and alveolar edema (Bai et al., 1999). In the peripheral lung and pleura, AQP1 is largely responsible for the osmotically-driven water movement across the membranes; an *ex-vivo* model where AQP1 was deleted showed a reduced water permeability (Yadav et al., 2020). However, AQP1 seems to have no significant effect on clearance of alveolar fluid in either adult or in newborn stage as shown in the knockout mice studies (Bai et al., 1999; Yadav et al., 2020). Upregulation of AQP1 was reported in lung injury induced in hypobaric hypoxia rodent models (Wang et al., 2016). AQP1 has also been shown to play a role in pulmonary hypertension (PH). For instance, targeting of AQP1 in hypoxia-induced PH ameliorated the effect of PH in treated mice (Schuoler et al., 2016). Acute exposure to Cl_2_ is known to cause pulmonary edema, hypoxemia, and pulmonary hypertension (Musah et al., 2017; de Genaro et al., 2021). Our results showing that AQP1 mRNA is differentially expressed in mice exposed to Cl_2_ are consistent with these findings and imply AQP1 role in the fluid infiltration (pulmonary edema) and respiratory distress that follows exposure to Cl_2_. An intervention by downregulating and/or inhibiting AQP1 will likely ameliorate these complications, which needs further investigation. However, further evaluation based on protein abundance and characterization is needed to support these functional conclusions. In this context, it has been reported that the correlation between transcription and protein expression may not always be absolute in eukaryotic genes (de Sousa Abreu et al., 2009; Maier et al., 2009; Vogel and Marcotte, 2012), including aquaporins (Prat et al., 2012; Bednar et al., 2015; Yepes-Molina et al., 2020). Nonetheless, in case of mammalian aquaporins, such disconnect between mRNA and protein levels has been reported to be dependent on the biological context (Bednar et al., 2015; Li et al., 2022). For instance, AQPs 1, 2, 3, 4, 5, and 6 in human renal carcinoma versus normal renal tissues showed consistent trends between their protein levels and mRNA levels (Li et al., 2022). In comparison, AQPs in human placental amnion versus reflected amnion showed similar qualitative profiles but discordant quantitative relationship for some AQP isoforms (Bednar et al., 2015).

In terms of histopathological changes, the Cl_2_ exposed lungs revealed interstitial widening, foamy macrophages, and debris material. Foamy macrophages were unique to the Cl_2_-exposed lungs among the different toxicity models. Any crosstalk between this phenotype and AQP1 needs further investigation. AQP1 has also been reported to promote the proliferation and migration of lung cancer cells *in vitro* and has been considered as a prognostic marker in lung adenocarcinoma (Wei and Dong, 2015; Yun et al., 2016). Though there was no compelling evidence of lung cell proliferation changes in the Cl_2_ toxicity model because of a short exposure or post-exposure period in this study, some reports do exist which are suggestive of a link between Cl_2_ exposure and lung adenocarcinoma (Vizcaya et al., 2013).

In contrast to AQP1, AQP3 transcriptional overexpression was observed in the chronic CNT toxicity model. Lack of significant upregulation of AQP3 due to Cl_2_ or CSE exposure indicated that this aquaporin is differentially responsive to particulate exposure and not to chemical exposure (acute or chronic). Primarily expressed in the basal epithelial cells of the nasopharynx and the large airways, AQP3 has been associated with Diffuse Alveolar Damage (DAD) and Acute Respiratory Failure (ARF) in humans (Pires-Neto et al., 2016) and shown to play a role in the triggering of inflammatory response in the lungs in ovalbumin-induced murine asthma (Ikezoe et al., 2016). This AQP is also reported to affect the membrane uptake of H_2_O_2_ which in turn affects cell signaling cascade in mammalian cells (Miller et al., 2010). The AQP3-mediated uptake of H_2_O_2_, is critical for chemokine-dependent T cell migration (Hara-Chikuma et al., 2012). This is in concordance with our published and current findings which showed that inhalation exposure to CNT nanoparticles induces inflammation (Frank et al., 2015; Frank et al., 2016; Frank et al., 2017) and upregulates AQP3 (current study) in exposed mouse lungs. This is also corroborated by the lung histology data which showed marked inflammatory changes in the CNT-exposed lungs in terms of infiltrated immune cells and granulomatous reactions.

Based on knock out studies, it has been reported that mice lacking in AQP3 and/or AQP4 show a reduced water permeability in the upper airway, but have little effect on the humidification of the airways, maintenance of the Airway Surface Liquid layer, and isomolar fluid absorption; however, AQP3 and AQP4 may have a prominent role to play in the osmotically driven water permeability in the airways (Song et al., 2000; Jayaraman et al., 2001; Yadav et al., 2020). Primarily expressed in the ciliated columnar cells in the nasopharyngeal, tracheal, and bronchial regions of the upper airways (Yadav et al., 2020), AQP4 was found to be affected by all the toxicant types albeit to a variable extent; the chronic CSE toxicity model showing a significant effect (*p* < 0.05) as compared to the other toxicity models. This may be indicative of an impacted osmotically driven water permeability in the airways over time though it warrants further evaluation based on AQP4 protein analysis.

It needs to be further considered that aquaporins, being complex proteins performing diverse functions, are also regulated by post-translational modifications such as phosphorylation (Nesverova and Tornroth-Horsefield, 2019). Furthermore, these transmembrane proteins are also regulated by “gating” thereby altering the water permeability rate through the pore itself, or by their “trafficking” between the plasma membrane and the intracellular storage vesicles (Kreida and Törnroth-Horsefield, 2015; Markou et al., 2022). Further evaluation in these post-translational aspects will yield deeper insights into the functional significance of the differentially regulated aquaporins.

Airway surface lining (ASL) is critical in maintaining lung homeostasis and function. ASL-associated proteins mucin and surfactant protein constitute an important component in the lung architecture and are critical players in maintaining respiration plasticity and innate defense in normal and exposed lungs. MUC5B is a major gel-forming mucin in mucus secreted from the proximal submucosal glands and the distal airway secretory cells (Rose and Voynow, 2006; Seibold et al., 2013; Nakano et al., 2016). It is associated with mucocilliary clearance and, when overexpressed, has been associated with idiopathic pulmonary fibrosis (IPF) (Evans et al., 2016), though the exact mechanism remains unknown. In case of IPF in human subjects, MUC5B is produced in cells lining distal airways and honeycomb cysts (Seibold et al., 2013; Nakano et al., 2016; Hancock et al., 2018). Notably, while acute Cl_2_ toxicity model induced high transcriptional upregulation (>20-fold) of the MUC5B, a downregulation was observed in the chronic exposure to chemical toxicants (CSE). A decreasing trend in MUC5B expression was also observed in chronic particulate toxicity models (CNT and CNT + CSE), though it was statistically insignificant. Recent reports have indicated Cl_2_ as a causative agent for airway-fibrosis (Musah et al., 2019). While an increase in the MUC5B expression may be indicative of an ensuing disease condition such as IPF or another pathological lung condition, a significant downregulation may also be indicative of poor mucocilliary clearance and a compromised innate immunoprotective function. The surfactant protein A (SP-A), known to be secreted by the alveolar type-II epithelial cells, has a significant role to play in reducing the surface tension at the alveolar air-liquid interface because of being a major protein component of the pulmonary surfactant material (Khubchandani and Snyder, 2001). The observed transcriptional downregulation of this gene, though variably in different toxicity models, may imply a suboptimal condition for gaseous exchange in the exposed lungs which may ultimately lead to other lung pathological conditions. The above inference on MUC5B and SP-A need to be further corroborated based on protein analysis especially in view of the reports that the downregulated eukaryotic genes may show discordance between the mRNA and protein levels (Vogel and Marcotte, 2012).

Lung epithelial integrity is one of the major factors which is often severely compromised when exposed to acute toxicity agents. The barrier function offered by the lung epithelium is dependent on its tight junction proteins. These proteins typically act as sealing agents between the adjacent epithelial cells (Schlingmann et al., 2015). A damage to these proteins would mean a breakdown of the lung epithelial barrier function, which in turn would be manifested in the form of secreted/leaked proteins in the BAL fluid (Holter et al., 1986; Wittekindt, 2017). A similar scenario may be expected in the endothelial cells which form a part of the lung epithelial-endothelial barrier. Our data show a significant upregulation in the expression of tight junction proteins particularly ZO-1 in the Cl_2_ exposed lungs which also showed an increase in the total protein content in the lumen (based on BAL fluid analysis), implying fluid infiltration. Though an upregulation response may seem like an anomaly, this is most likely a compensatory response of the cells to the damaged epithelial or endothelial barrier (as reflected by an increase in the protein infiltration in the BAL fluid). Moreover recent studies also suggest that ZO-1 is even more critical in the effective mucosal repair when compared to its role in the barrier function (Kuo et al., 2021). This may also explain the overexpression of ZO-1 as a response to the mucosal damage caused by acute exposure to Cl_2_, a corrosive toxicant. Nonetheless, these functional extrapolations need to be corroborated by further analysis of these targets.

## Conclusion

To our knowledge, this is the first demonstration of lung aquaporins as molecular targets of inhaled study toxicants (particulates and chemicals) in different exposure type scenarios (acute versus chronic). Notably, AQP1 and AQP3 were differentially upregulated in acute Cl_2_ exposed and chronic CNT exposed lungs, respectively. The toxicant-specific response of these selected AQPs, implied their potential as differential markers of toxicant exposure. The study further revealed corresponding patterns in expression of other pathophysiological targets including the lung mucosal proteins MUC5B and SP-A (responsible for homeostasis and innate lung lining defense) as well as the tight junction proteins ZO-1 and OCLN (responsible for the epithelial-endothelial barrier function of the lungs). Further investigations should focus on protein quantification, localization, and post translational modifications of these targets. Taken together, these findings lay the foundation for developing specific aquaporins as toxicant-type specific exposure biomarkers as well as potential targets for therapeutic interventions in acute versus chronic exposures to emerging and chemical threat/warfare-relevant toxicants.

## Data Availability

The original contributions presented in the study are included in the article/Supplementary Materials, further inquiries can be directed to the corresponding author.
