# Supplementary figures and images for: Differential modulation of lung aquaporins among other pathophysiological markers in acute (Cl2 gas) and chronic (carbon nanoparticles, cigarette smoke) respiratory toxicity mouse models
Source: Front Physiol. 2022 Sep 28;13:880815. doi: 10.3389/fphys.2022.880815 (PMC9554232; doi:10.3389/fphys.2022.880815)

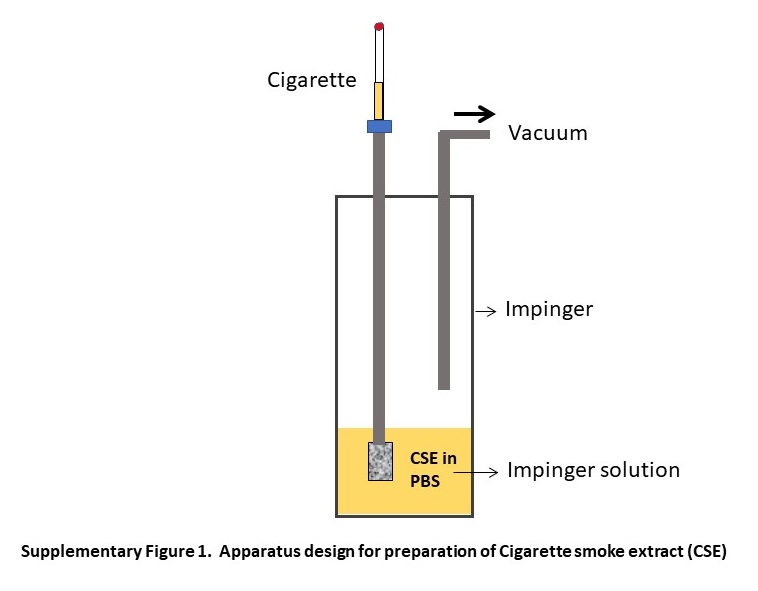

Supplement: Supplementary file 1 [file Image1.JPEG]

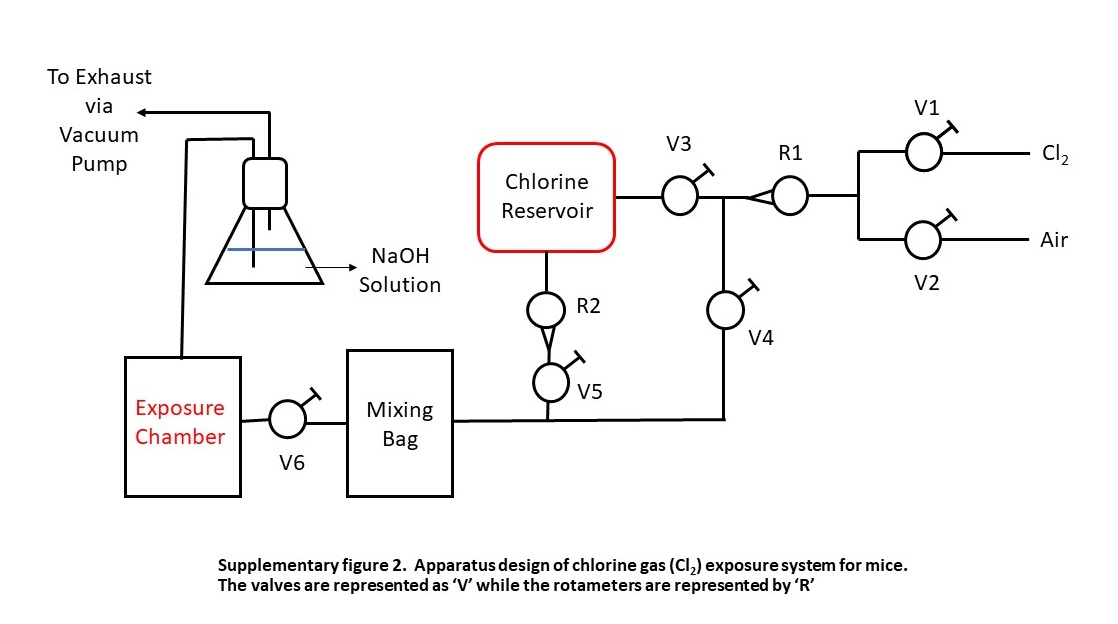

Supplement: Supplementary file 2 [file Image2.JPEG]
